# Case Report: Diagnosis of Primary *Klebsiella pneumoniae* in Cervical Spine by Metagenomic Next-Generation Sequencing

**DOI:** 10.3389/fsurg.2022.800396

**Published:** 2022-03-15

**Authors:** Tao Li, Qile Gao, Chaofeng Guo, Yanbing Li

**Affiliations:** ^1^Department of Spine Surgery and Orthopaedics, Xiangya Hospital, Central South University, Changsha, China; ^2^National Clinical Research Center for Geriatric Disorders, Xiangya Hospital, Central South University, Changsha, China; ^3^Department of Clinical Laboratory, Xiangya Hospital, Central South University, Changsha, China

**Keywords:** *K. pneumoniae*, cervical infection, metagenomic next-generation sequencing (mNGS), operation, diagnosis

## Abstract

**Introduction:**

Spinal infection is a disease that affects the intervertebral disks or adjacent paravertebral tissue in the vertebral body. There are few reports of spinal infections caused by *Klebsiella pneumoniae*. Cervical spine infection by *K. pneumoniae* especially preoperative is extremely rare. Nowadays, metagenomic next-generation sequencing (mNGS) has led to the accurate and timely diagnoses of numerous infectious diseases.

**Case Presentation:**

We described a case of a 64-year-old woman, with a chief complaint of neck, shoulder, and upper limb pain for 10 days. The patient had symptoms of abscess compression before surgery, and inflammatory indicators such as erythrocyte sedimentation rate (ESR), C-reactive protein (CRP), and procalcitonin (PCT) were significantly elevated. The patient's imaging suggested cervical infectious lesions, and the patient had no symptoms of tuberculosis poisoning, and the blood samples associated with tuberculosis were negative. The patient was diagnosed with cervical suppurative infection before surgery. For the patient who failed conservative treatment and had abscess compression, we performed anterior cervical surgery to remove the lesion at an early stage and collected intraoperative specimens for culture and mNGS. Postoperative antibiotic treatment was adjusted according to the etiology and drug sensitivity.

**Conclusion:**

This case suggests that the clinical symptoms of *K. pneumoniae* infection are not typical and the imaging examination lacks specificity. When the clinical diagnosis of etiology is not clear or there are symptoms such as abscess compression, early surgical specimens can be collected for culture and mNGS to identify the pathogen, and postoperative sensitive antibiotics can be used to continue treatment. This helps to identify the cause as early as possible, treat it effectively early, relieve symptoms, prevent complications, and keep the spine stable.

## Introduction

Spinal infection is a disease that affects the intervertebral disks or adjacent paravertebral tissue in the vertebral body (1). It is usually caused by bacterial microorganisms and requires a high level of diagnosis and treatment, in most cases requiring a multidisciplinary approach by spinal surgeons, radiologists, and infectious disease specialists (2). The most common pathogen of spinal infection is *Staphylococcus aureus*, followed by *Streptococcus* and *Enterococcus* (3). *Klebsiella pneumoniae* is a common pathogen of nosocomial infections, yet there are few reports of spinal infections caused by *K. pneumoniae* (4, 5). Cervical infection by *K. pneumoniae* especially preoperative is extremely rare. We recently admitted a patient with primary cervical *K. pneumoniae* infection, whose pathogens were first identified by metagenomic next-generation sequencing (mNGS) and treated surgically.

## Case Presentation

**Figure 1:**
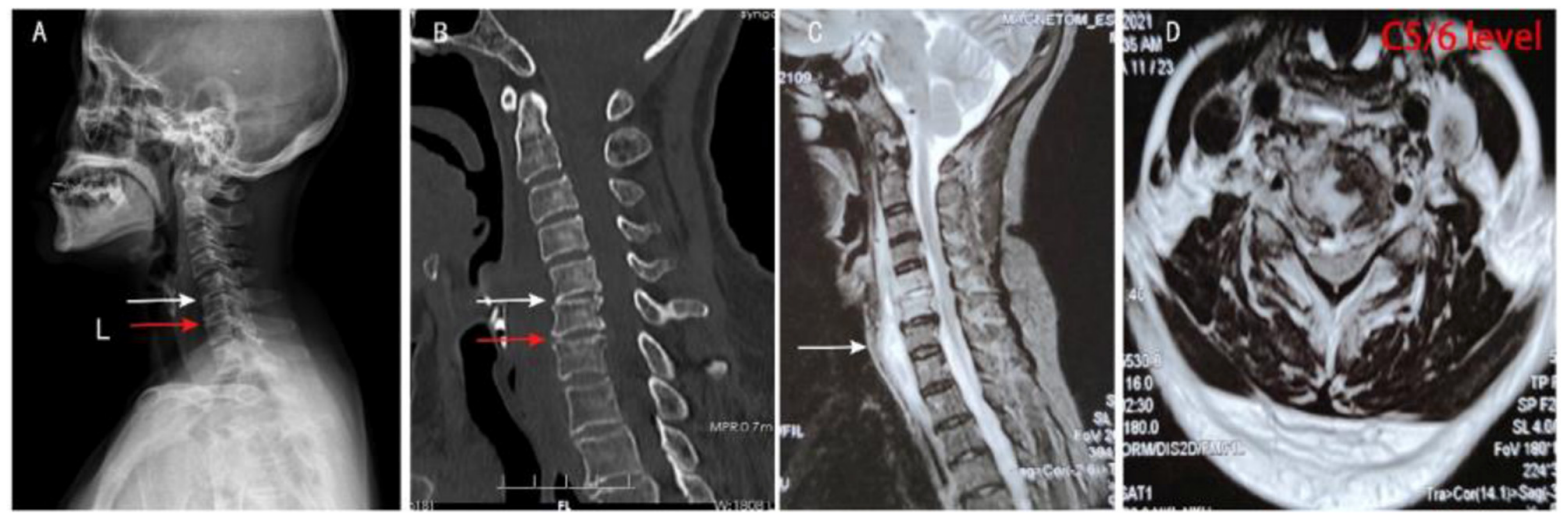
Preoperative imaging results of the patient. **(A,B)** Preoperative cervical spine X-ray and CT showed narrowing of the C5/6 (white arrow) and C6/7 (red arrow) intervertebral space, poor bone structure at the upper and posterior edges of the C6 vertebral body, and thickened soft tissue at the anterior edge of the C4-T2 vertebral body. **(C,D)** Preoperative sagittal and transverse MRI of the cervical spine showed the abscess was located in front of the C2-T2 vertebral body (white arrow) and also in the C5/6 intervertebral space, both C5 and C6 vertebral bodies showed hypersignal changes.

The patient was a 64-year-old woman, with a chief complaint of neck, shoulder, and upper limb pain for 10 days. On February 19, 2021, the patient was admitted to our spinal surgery ward. Furthermore, the patient had painful difficulty in swallowing, neck and upper limbs pain which were not relieved by rest or sleep. This made the patient take painkillers daily before sleep. The patient denies any history of night sweats, tiredness, cough, loss of power in the upper limbs, tingling, numbness, and loss of appetite. The examination showed clear breathing sounds on both lungs, and no fistula or subcutaneous mass on the neck. Furthermore, the patient had the following specific signs: diminished cervical lordosis, painful palpation at C4–6 supraspinous and paravertebral muscle, pain radiating in both upper limbs and at the back, and moderate limitation on both flexion and extension of the neck. On the other hand, the patient exhibited muscle strength in the upper limbs grades 4 and normal muscle strength in the lower limbs, as well as normal sensation and muscle tone on both limbs. Laboratory investigation showed amounts of white blood cells (WBC) at 10.3 × 10^9^/L, neutrophil count percent (NEUT%) at 82.7%, procalcitonin (PCT) at 0.135 ng/ml, erythrocyte sedimentation rate (ESR) at 104 mm/h, and C-reactive protein (CRP) at 67.6 mg/L (Table 1). The patient had a maximum fever of 38.3°C, on hospitalization, but was normalized after 1 day of treatment. Tuberculin skin test (TST), anti-tuberculosis antibody test, and T cell antigen-specific (ESAT-6 and CFP-10) IFN-γ release assays (IGRAs) were normal. Continuity morning sputum direct smear for three times resulted negative, tuberculosis infection T cells result negative and three consecutive automatic acid-fast staining results negatives. Radiological examination showed that cervical spine X-ray reported degenerative changes at C5/6, as well as C6/7 intervertebral space (Figure 1A). Cervical and lung plain CT scan indicated soft tissue thickening at the anterior margin of the C4-T2 vertebra, narrowing of the C5/6 intervertebral space (Figure 1B). Cervical MRI plain scan revealed disc herniation at C3/4, C4/5, C5/6, C6/7, and showed the abscess was located in front of the C2-T2 vertebral body and also in the C5/6 intervertebral space. Moreover, the C5 and C6 vertebral bodies showed hypersignal changes (Figures 1C,D). We had a ward diagnosis of suppurative infection of the cervical spine with a differential of cervical tuberculosis. The patient was treated with intravenous piperacillin/tazobactam 4.5 g every 8 h for 3 days and was then planned for surgery.

**Table 1:** Patient treatment timeline and relevant main clinical data.

	**First 3 days after admission**	**Day 3 (Day of surgery)**	**One weeks after surgery**	**Seven weeks after surgery**	**3 month after surgery**
Therapy method	Intravenous antibiotics	Surgical treatment	Intravenous antibiotics	Intravenous antibiotics	Oral antibiotics for 5 weeks
Clinical manifestations	Had painful difficulty in swallowing, neck and upper limbs pain	—	No fever, mild neck and upper limb pain	No special discomfort	No special discomfort
WBC (× 10^9^/L)	10.3	—	Normal	Normal	Normal
NEUT%	82.7%	—	78.2%	Normal	Normal
ESR (mm/h)	104	—	120	107	Normal
CRP (mg/L)	67.6	—	25.7	11.36	Normal
PCT (ng/mL)	0.135	—	Normal	Normal	Normal

Surgery was done under general endotracheal anesthesia; the patient was positioned supine and a standard Smith-Robinson approach for anterior exposure of cervical spine was done on the right side. After routine exposure, the anterior esophageal fascia was found inflamed and when the prevertebral fascia was cut open light-yellow pus was found around C5/6 intervertebral disc. The same intervertebral space was found necrotic with pus. The pus was drained and thorough debridement and decompression were done, all diseased tissues and necrotic disc were removed. The incision site was washed with hydrogen peroxide followed by normal saline. During the operation, the sample was taken for rapid histological examination and reported C5/6 inflammatory lesions. The titanium mesh filled with allograft was firmly inserted the C5/6 intervertebral space. A titanium plate of appropriate length was placed in front of C5 and C6 vertebral bodies, locked firmly with screws. C-arm fluoroscopy was used to confirm the proper positioning of the implants. Somatosensory evoked potential (SEP) and motor evoked potential (MEP) were used intraoperatively to check the occurrences of new neurological deficits. Intraoperative diseased lesions were collected and sent for culture and other pathological examinations. Twenty hours after surgery, mNGS detected *K. pneumoniae* (sequence number 3824), and the drug-resistant gene SHV-9 (sequence number 1), which implied resistance to β-lactam antibiotics. After consulting experts from the infectious department, the decision to treat the patient with intravenous piperacillin/tazobactam 4.5 g every 8 h. Blood culture results at the local hospital also showed *K. pneumoniae* 3 days after surgery, suggesting that *K. pneumoniae* is sensitive to piperacillin/tazobactam. The results of mNGS were confirmed by tissue bacterial culture 1 week after surgery.

**Figure 2:**
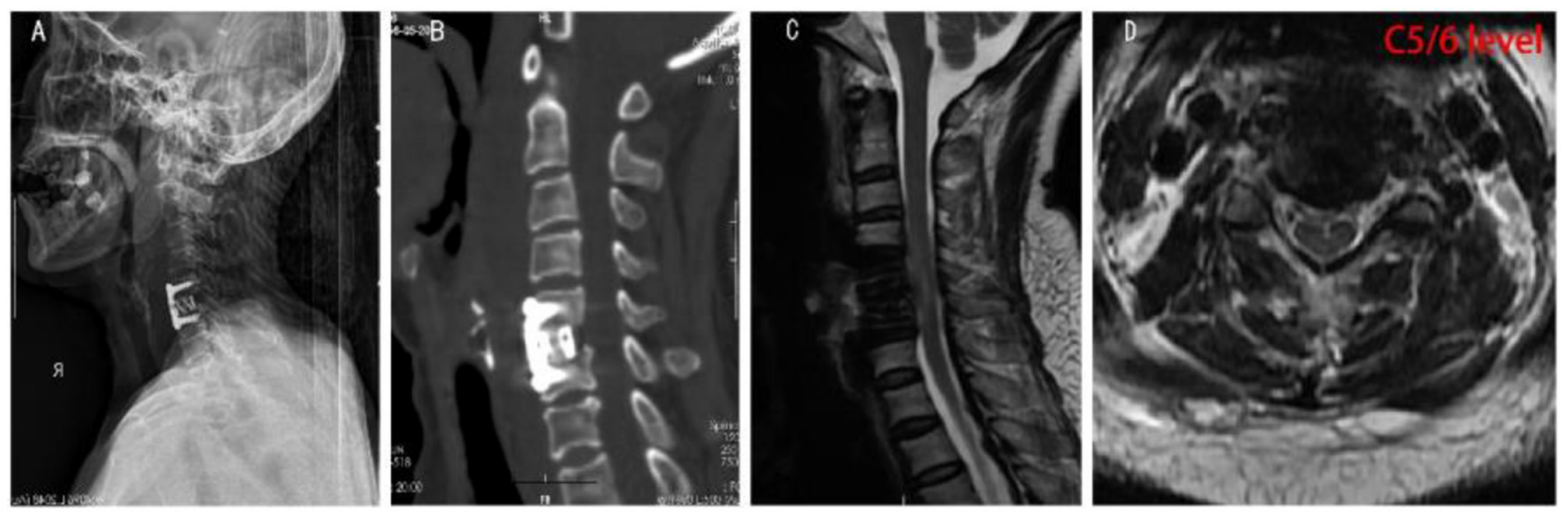
Postoperative imaging results of the patient. **(A,B)** Postoperative X-ray and CT of the cervical spine showed changes in the C5-6 vertebral body after screw internal fixation, no slippage and fracture were observed, and cage shadow was observed in the C5/6 intervertebral space. **(C,D)** Postoperative sagittal and transverse MRI of the cervical spine showed internal fixation shadow in the C5-6 vertebrae, showing postoperative changes, with internal fixation in place. No obvious effusion was observed in the C2-T2 prevertebral space.

The DNA test result for the Mycobacterium tuberculosis complex group was negative, but both anaerobic and aerobic cultures of bone tissues produced *K. pneumoniae*, which further confirmed the diagnosis of *K. pneumoniae* infection. To find the source of the pathogen, sputum smear and culture were performed again, but no *K. pneumoniae* was found. One week after surgery, imaging showed no loosening, fracture, prolapse, or displacement of the internal fixation devices, and the titanium cage was in a good position (Figure 2). Postoperative histopathology revealed a large number of inflammatory cells exudate, suggesting chronic suppurative inflammation of the C5/6 disc (Supplementary Figure 1).

One week postoperative the patient's, general condition improved, neck and shoulder pain alleviated, inflammatory markers PCT returned to normal value, NEUT% 78.2%, ESR 120 mm/h, CRP decreased from 67.6 to 25.7 mg/L, and the patient showed no symptoms of fever. The patient was discharged from our hospital and continued with the same antibiotic treatment for 6 weeks in the nearby local hospital (7 weeks after surgery), wherein WBC and PCT returned to normal at re-examination, with CRP at 11.36 mg/L and ESR at 107 mm/h. Oral levofloxacin was continued for 5 weeks (3 months after surgery), wherein WBC, ESR, CRP, and PCT were normal (Table 1). During follow-up, there were no postoperative limb pain, decreased muscle strength, numbness, and other spinal cord and nerve injuries.

## Discussion

*Klebsiella pneumoniae* is among the opportunistic pathogens that mainly cause hospital-related infections as well as a risk factor for serious community-acquired infections (6, 7). The most common sites of infection are the urinary tract and respiratory tract, and rarely attack the vertebral column. In addition, it has been reported that some *K. pneumoniae* produce an extended-spectrum β-lactamase, which can develop resistance to β-lactamide antibiotics (8). The widespread use of broad-spectrum antibiotics weakened immunity and other factors that can easily cause spinal infections (9).

Culture is the gold standard for the identification of pathogenic agents. However, 2–3 days are typically required for initial results and up to 1 week for confirmation (10). In recent years, a large number of studies have reported that mNGS is a novel method to detect pathogens of infectious diseases with high speed, specificity, and sensitivity (11). mNGS only needs 24–48 h to give the final result after receiving the sample, which is determined by the sequencing technology, modes, and bioinformatics software used by the clinician (12). Therefore, mNGS can detect pathogens early and guide clinical treatment faster than traditional culture.

The treatment of spinal infection includes conservative treatment and surgical treatment. Conservative treatment mainly consists of a large dose of broad-spectrum antibiotics, which lasts for a long time. At present, some scholars advocate active surgical treatment, complete removal of lesions, prevention of complications such as infection spread, and postoperative adjustment of antibiotics according to culture and drug sensitivity tests (13, 14). The best treatment for infection with abscess formation involves surgical decompression and drainage, and antibiotic treatment for up to 12 weeks (15). Clinically, for patients with spinal infection complicated with bone destruction, the treatment principle is mainly supplemented by surgery and anti-tuberculosis chemotherapeutic drugs. The focus of spinal infection was removed by operation, the balance of the sagittal plane of the spine was reconstructed by the internal fixation system, and bone graft was grafted in the focus area in order to achieve long-term bony fusion and recovery of spinal stability (16). In this case, the abscess was located in front of the cervical vertebra and also in the C5/6 intervertebral space, and the intervertebral space infection was mainly located in the anterior column of the spine. Therefore, anterior surgery was more effective in removing the lesion completely, and a full course of antibiotics was given after surgery.

Extrapulmonary infection of *K. pneumoniae* is rare in clinical practice, and cervical *K. pneumoniae* infection is even rarer in clinical practice. The patient had obvious symptoms of abscess compression before surgery, and inflammatory indicators such as PCT and CRP were significantly increased. The patient's imaging suggested infectious lesions, and the patient had no symptoms of tuberculosis poisoning such as afternoon low fever, fatigue and night sweats.In addition, the blood samples associated with tuberculosis were negative, so tuberculosis infection was not considered before the operation, and anti-infection treatment was mainly applied. After the operation, mNGS found *K. pneumoniae* within 24 h, later, traditional culture confirmed the diagnosis of cervical *K. pneumoniae* infection. We need to consider that it was the hematogenous or transesophageal route, even if it is asymptomatic elsewhere. After a detailed examination of the medical history, the patient showed no high risk of infection, no *K. pneumoniae* was found in sputum smear and culture, and no infection was found in other parts of the body. Therefore, it is highly likely that *K. pneumoniae* originated in the cervical vertebra. In the case of cervical spine infection combined with prevertebral space effusion, the patient had symptoms caused by abscess compressing on adjacent organs, and these were the indications for early operation, so surgical treatment was used to remove the lesions and postoperative antibiotic treatment lasted for a long time according to the results of drug sensitivity. The standardized intravenous treatment of sensitive antibiotics greatly shortened the treatment course. During the postoperative follow-up of 3 months, the patient's discomfort symptoms such as neck and shoulder pain and dysphagia were disappeared, and the stability of the cervical spine was restored.

## Conclusion

This case report implies that the clinical symptoms of *K. pneumoniae* infection are not typical, and the imaging examination lack specificity. When the disease is highly suspected clinically or there are symptoms of abscess compression, mNGS can be taken to identify the pathogen within hours, and sufficient treatment can be performed by intravenous treatment with sensitive antibiotics combined with surgical removal of the lesion. We found this beneficial to eradicate the disease, relieve the symptoms, and maintain the stability of the spine as early as possible.

## Data Availability Statement

The datasets presented in this article are not readily available due to ethical and privacy restrictions. Requests to access the datasets should be directed to the corresponding authors.

## Ethics Statement

The studies involving human participants were reviewed and approved by Ethics Committee of Xiangya Hospital, Central South University. The patients/participants provided their written informed consent to participate in this study. Written informed consent was obtained from the individual(s) for the publication of any potentially identifiable images or data included in this article.

## Author Contributions

TL wrote this case. QG proofread the text. CG and YL revised the report according to journal requirements. All authors have read and approved the manuscript.

## Funding

This work was supported by the National Natural Science Foundation of China [grant numbers 82072460 and 82170901] and the Natural Science Foundation of Hunan Province, China [grant numbers 2019JJ40525 and 2019JJ40523].

## Conflict of Interest

The authors declare that the research was conducted in the absence of any commercial or financial relationships that could be construed as a potential conflict of interest.

## Publisher's Note

All claims expressed in this article are solely those of the authors and do not necessarily represent those of their affiliated organizations, or those of the publisher, the editors and the reviewers. Any product that may be evaluated in this article, or claim that may be made by its manufacturer, is not guaranteed or endorsed by the publisher.

## Supplementary Material

Supplementary Figure 1 Pathological results of the patient. **(A,B)** The pathological section of the C5/6 intervertebral disc showed a large number of inflammatory cells exudate, suggesting chronic suppurative inflammation.
