# Supplementary figures and images for: Case Report: Diagnosis of Primary Klebsiella pneumoniae in Cervical Spine by Metagenomic Next-Generation Sequencing
Source: Front Surg. 2022 Mar 15;9:800396. doi: 10.3389/fsurg.2022.800396 (PMC8964782; doi:10.3389/fsurg.2022.800396)

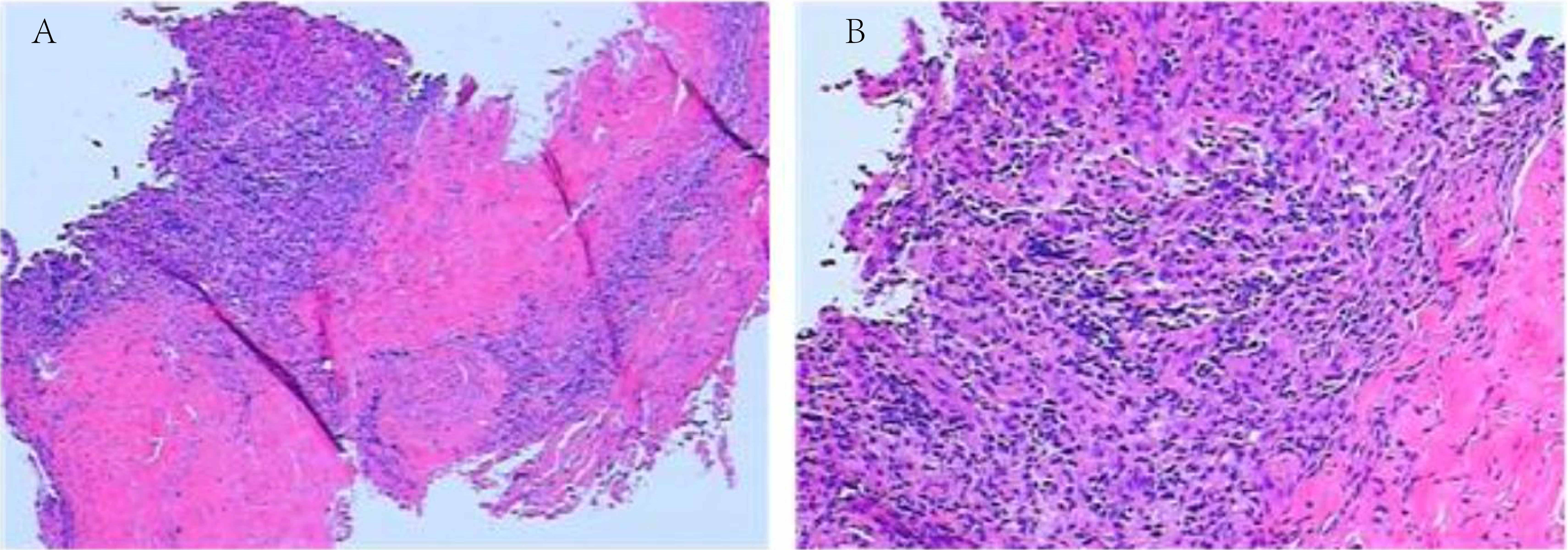

Supplement: Supplementary Figure 1 — Pathological results of the patient. (A,B) The pathological section of the C5/6 intervertebral disc showed a large number of inflammatory cells exudate, suggesting chronic suppurative inflammation. [file Image_1.JPEG]
